# Endothelial cell in tumor angiogenesis: Origins, mechanisms, and therapeutic implication

**DOI:** 10.1016/j.gendis.2025.101611

**Published:** 2025-03-24

**Authors:** Yulong Han, Binqiang Zhu, Shu Meng

**Affiliations:** a State Key Laboratory of Respiratory Disease, The First Affiliated Hospital of Guangzhou Medical University, Guangzhou Medical University, Guangzhou, Guangdong 510120, China; b Department of Basic Science Research, Guangzhou National Laboratory, Guangzhou, Guangdong 510005, China

**Keywords:** Angiogenesis, Anti-angiogenic therapy, Origins of endothelial cells, Tumor angiogenesis, Tumor endothelial cells

## Abstract

A hallmark feature of cancer is its capacity to induce the development of new blood vessels. Anti-angiogenic therapies are commonly employed to combat various types of cancer. Despite notable advancements in this field, limited efficacy and resistance remain critical challenges. While anti-angiogenic therapy primarily targets endothelial cells within the abnormal vasculature, the origins of tumor vascular endothelial cells in solid tumors remain a subject of ongoing debate. Unraveling the origins of these endothelial cells is crucial for developing more effective strategies to combat tumor angiogenesis. This review summarizes the latest findings on the origins of endothelial cells in tumor angiogenesis and explores the progress, limitations, and future directions of anti-angiogenic therapy.

## Introduction

In the 1970s, Dr. Folkman proposed the hypothesis that tumor growth relies on the blood vessels.^1^ He also identified a tumor-derived factor, suggesting that blocking angiogenic signaling could inhibit new blood vessel formation and induce tumor dormancy.^2^ This groundbreaking discovery challenges the traditional view that intra-tumor vasculature is purely inflammatory and not essential for tumor growth. Meanwhile, this discovery also lays a solid foundation for anti-tumor therapies targeting angiogenesis, highlighting the critical role of tumor angiogenesis.^3^

The accelerated proliferation of tumor cells requires a constant supply of oxygen and nutrients. Consequently, tumor cells secrete various pro-angiogenic factors to stimulate angiogenesis. However, an imbalance between pro- and anti-angiogenic signals leads to abnormal vascular structure and impaired function. Endothelial cells (ECs) in these abnormal vasculatures emerge as the primary targets of anti-angiogenic therapy. Despite remarkable strides in anti-angiogenic therapy, the precise origins of tumor vascular ECs within solid tumors remain debated. Therefore, unraveling the origins of these ECs is of paramount significance in the pursuit of more effective strategies to combat tumor angiogenesis.

This article reviews the origins of ECs in angiogenesis and the recent advancements in anti-angiogenic therapy. A comprehensive understanding of the origins of tumor vascular ECs and progress in anti-angiogenic therapy may reveal novel cellular or molecular targets to block oxygen and nutrient supply, potentially enhancing the clinical treatment of solid tumors.

### Hypoxic angiogenesis

Angiogenesis refers to the formation of new vessels through the sprouting and remodeling of existing blood vessels. This process requires the coordinated interaction of multiple cells, cytokines, and growth factors and occurs in response to various physiological and pathological stimuli.^4^, ^5^, ^6^ Under hypoxic conditions, angiogenesis follows an organized pattern characterized by pericyte coverage, regular branching, and a stratified vascular architecture.^7^

There are two major mechanisms involved in hypoxia-driven angiogenesis. The first one is sprouting angiogenesis, which is defined by the proliferation and migration of endothelial tip cells. These tip cells extend protrusions and navigate toward hypoxic regions, where they initiate the formation of new capillaries. The sprouting process involves a coordinated interplay between ECs, pericytes, and extracellular matrix components to establish functional vascular networks.^8^^,^^9^

The second mechanism is intussusception angiogenesis, an important alternative and complementary form of sprouting angiogenesis. In this process, existing blood vessels are divided into two by the insertion of tissue pillars, facilitating rapid vascular expansion and remodeling by creating new vascular channels within the existing vascular bed.^10^, ^11^, ^12^, ^13^, ^14^, ^15^, ^16^, ^17^, ^18^ This process is particularly important in the adaptation to hypoxia, as it enables the efficient redistribution of blood flow to areas with increased metabolic demand.

Overall, angiogenesis is a complex and dynamic process that involves multiple cellular and molecular events. Understanding the mechanisms underlying hypoxia-driven angiogenesis is essential for developing therapeutic strategies aimed at modulating vascular growth and remodeling in various pathological conditions, including ischemic diseases and cancer.

### Tumor angiogenesis

Tumor angiogenesis is crucial for tumor progression, metastasis, and invasion.^19^, ^20^, ^21^, ^22^ Tumor vasculature often exhibits aberrant structure and function,^23^ characterized by irregular tortuosity, severely disorganized endothelium, poorly organized pericyte coverage, and increased permeability.^24^, ^25^, ^26^, ^27^ These anomalous vascular features not only facilitate tumor dissemination and metastasis but also hinder immune cell infiltration and compromise the delivery of therapeutic agents.^28^, ^29^, ^30^, ^31^, ^32^

In the early stage of tumor development, the tumor exists in an avascular state, with a volume typically less than 2 mm^3^, where intra-tumoral vascularization is minimal.^33^ However, as the tumor grows beyond this size threshold, it transitions into a vascularized state characterized by robust neovascularization to support its expanding metabolic demands. During this phase, ECs become disconnected, resulting in elevated interstitial pressure, increased vascular permeability, and impaired blood perfusion.^34^, ^35^, ^36^, ^37^, ^38^, ^39^, ^40^, ^41^, ^42^, ^43^

Even though sprouting angiogenesis is the principal mechanism of new vessel formation, tumors also utilize various other mechanisms to promote angiogenesis. One such mechanism is the intussusceptive process, characterized by the insertion of interstitial tissue pillars into the lumen of preexisting vessels, splitting them into two new functional vessels.^44^ Tumors also exploit vessel mimicry, where tumor cells create vessel-like channels surrounding normal blood vessels to enhance oxygen and nutrient supply.^45^, ^46^, ^47^ Another strategy is vessel co-option, in which tumor cells hijack nearby blood vessels from the surrounding tissue to sustain their growth.^48^, ^49^, ^50^ Additionally, the differentiation of cancer cells, especially cancer stem cells, into ECs contributes to the formation of new blood vessels.^14^ Endothelial progenitor cells (EPCs) from the bone marrow can differentiate *in situ* and incorporate into growing vessels to facilitate rapid neovascularization.^44^ Collectively, these mechanisms drive the angiogenic switch, thereby promoting tumor progression.

### Key molecules and signaling pathways in tumor angiogenesis

Decades of extensive mechanistic research have identified critical molecules and signaling pathways in tumor angiogenesis, significantly advancing our understanding of this intricate process. The angiogenic system consists of a wide variety of molecules, including growth factors, such as fibroblast growth factor (FGF), vascular endothelial growth factor (VEGF), platelet-derived growth factor (PDGF), angiopoietin (ANG), adhesion molecules such as integrins and cadherins, proteases like matrix metalloproteinases (MMPs), and extracellular matrix proteins like fibronectin and collagen. Additionally, transcription factors such as the hypoxia-inducible factor (HIF) and some signaling molecules like mammalian target of rapamycin (mTOR), AKT (protein kinase B), p38 mitogen-activated protein kinase (MAPK), and nitric oxide (NO) play crucial roles in orchestrating angiogenesis. Furthermore, biomolecules like thrombospondin-1, angiostatin, endostatin, and interleukins also contribute to regulating angiogenic processes.^51^ These diverse biomolecules initiate downstream signaling cascades that modulate the expression of related genes, ultimately promoting EC proliferation, survival, and angiogenesis. The intricate crosstalk between these signaling pathways underscores the complexity of tumor angiogenesis, emphasizing the diverse and multifactorial nature of its regulation.

The FGF family is the first group of growth factors identified as being associated with angiogenesis. FGF1, a member of the acidic fibroblast growth factor family, promotes the proliferation and differentiation of vascular cells. FGF2, also referred to as bFGF, is considered the most prominent pro-angiogenic factor within the FGF family, actively promoting various angiogenesis-related processes.^52^ Upon activation by FGFs, fibroblast growth factor receptors (FGFRs) trigger downstream signaling cascades, including the Ras-Raf1-MAPK and phosphoinositide 3-kinase (PI3K)-AKT pathways, leading to EC proliferation. Additionally, the Janus kinase (JAK)-signal transducer and activator of transcription (STAT) pathway facilitates cell migration and invasion, while the phospholipase C gamma (PLCγ)-protein kinase C (PKC) pathway regulates cell migration.^53^^,^^54^ The FGF/FGFR axis promotes the coupling of p120-cadherin with VE-cadherin, stimulates the shedding of MMP2 and MMP9 from the cell surface, and controls c-MYC/hexokinase 2 (HK2) pathways, collectively promoting angiogenesis.^55^

The VEGF family serves a central role in the process of angiogenesis. This family is composed of seven members, including VEGFA, VEGFB, VEGFC, VEGFD, placental growth factor (PlGF), and the non-human genome-derived VEGFE and svVEGF. Among them, VEGFA stands out as the key regulator of angiogenesis, playing a critical role in promoting tumor growth, proliferation, invasion, metastasis, and drug resistance.^56^ The VEGFA/VEGFR2 signaling pathway actively regulates angiogenesis by activating downstream pathways such as the PLCγ-PKC-mitogen-activated protein kinase kinase (MEK)-Extracellular regulated protein kinase (ERK) pathway.^56^^,^^57^ VEGFA/VEGFR2 activates the Raf/MEK/ERK pathway, which promotes cell proliferation and vascular permeability,^57^^,^^58^ and it also activates the Src-PI3K-Akt pathway, enhancing cellular activity and promoting cell survival. VEGFA stimulation of VEGFR2 triggers the p38 MAPK and FAK pathways, promoting EC migration.^52^^,^^56^, ^57^, ^58^, ^59^, ^60^, ^61^ Furthermore, ligand binding to VEGFR2 triggers the activation of the Ras pathway, which intensifies vascular tube formation.^58^

The PDGF family functions as a crucial mitogen for a range of mesenchymal cell types, including fibroblasts, smooth muscle cells, and glial cells. PDGF plays a crucial role in regulating cell growth, differentiation, angiogenesis, and pericyte recruitment through paracrine and autocrine signaling pathways.^62^ PDGFA promotes cell proliferation, differentiation, metastasis, invasion, and angiogenesis. PDGFB has significant cancer-driving efficacy.^52^ The interaction between PDGFB and its receptor, PDGFRβ, is vital for signaling cascades that regulate various cellular processes. Specifically, the PDGFB/PDGFRβ interaction activates downstream signaling cascades, including the MAPK/ERK, PI3K/AKT, and JNK pathways, which are essential for vascular maturation, pericyte recruitment, and VEGF expression.^62^, ^63^, ^64^ Additionally, PDGFDD binds to PDGFRβ and promotes angiogenesis by activating the Notch1/Twist1 pathway.^65^ PDGFDD/PDGFRβ axis also stimulates EC proliferation by inducing AKT phosphorylation.^65^

ANG and Tie signaling systems are critical for blood vessel formation and maintenance.^66^, ^67^, ^68^ Endothelial TIE1 (tyrosine kinase with immunoglobulin-like and EGF-like domains 1) does not bind ANG1 or ANG2 but predominantly interacts with TIE2.^69^ ANG1 and ANG2 play opposing roles in angiogenesis regulation. ANG1, upon binding to and activating the TIE2 receptor, suppresses angiogenesis by controlling the Akt/survivin pathway and influencing perivascular-EC interactions and endothelial survival.^30^^,^^70^^,^^71^ However, recent studies have shown that the loss of TIE1 enhances Notch signaling, suggesting TIE1 is a positive regulator of tumor angiogenesis.^69^ Conversely, ANG2, mainly produced by ECs, acts as an endogenous antagonist of ANG1, enhancing angiogenesis by competitively binding to TIE2 and integrin receptors, promoting vascular remodeling and sprouting.^30^^,^^72^, ^73^, ^74^

The receptor tyrosine kinase (RTK) family, which includes Eph receptors and ephrin ligands, plays a crucial function in tumor angiogenesis, progression, and metastasis. The mammalian Eph system comprises 14 receptor tyrosine kinases (9 EphA and 5 EphB receptors).^75^ Up-regulation of the Eph-ephrin signaling pathway is observed in tumor angiogenesis. Specifically, the Ephrin B2/Eph receptor B4 (EphB4) signaling pathway signaling pathway plays a vital role in sprouting, vascular maturation, and revascularization during tumor angiogenesis and is also integral to the VEGF-Delta-like ligand 4 (Dll4)/Notch-Ephrin B2 pathway.^76^, ^77^, ^78^, ^79^ Recent studies have revealed additional roles of the Eph system in tumor vascularization. For instance, Eph is found to collaborate with VEGF to regulate the downstream JNK/PI3K pathway. EphA2 mediates angiogenesis through its association with cluster of differentiation 44 (CD44) and SRC, while EphB4 promotes tumor angiogenesis by inhibiting the Ras pathway.^80^, ^81^, ^82^

In addition to the factors mentioned above, various other molecules contribute to angiogenesis. Transcription factor HIF, activated under low oxygen conditions, stimulates the expression of pro-angiogenic factors such as VEGF, FGF, and PDGF, which promotes angiogenesis via MAPK, PI3K, PLCγ, STAT, ERK1/2, receptor for advanced glycation end-products (RAGE), and endothelial nitric oxide synthase (eNOS) pathways.^83^, ^84^, ^85^ MMPs remodel the extracellular matrix, facilitating new blood vessel sprouting and growth. Transforming growth factor beta (TGFβ) activation induces VEGF expression through MMP-mediated mechanisms, thereby promoting angiogenesis.^86^^,^^87^ Furthermore, TNFα, produced by macrophages, regulates angiogenesis by modulating VEGFR2 expression and inducing MMP9, which contributes to angiogenesis.^88^^,^^89^ Pleiotrophin, a protein implicated in promoting angiogenesis across various cancer types, regulates VEGF expression by binding to receptors protein tyrosine phosphatase receptor zeta (PTPRζ) or anaplastic lymphoma kinase (ALK).^90^^,^^91^ Collectively, these findings provide deeper insights into the complex mechanisms underlying tumor angiogenesis and cancer progression.

### Origins of ECs in hypoxic angiogenesis

ECs are pivotal for angiogenesis, yet the precise source of these cells remains an unresolved issue.^92^ Understanding the origin of ECs is crucial for elucidating neovascularization processes and developing targeted therapies. Angiogenesis under physiological conditions is precisely regulated and highly coordinated, following a specific pattern to ensure tissue function and repair.

**Figure 1:**
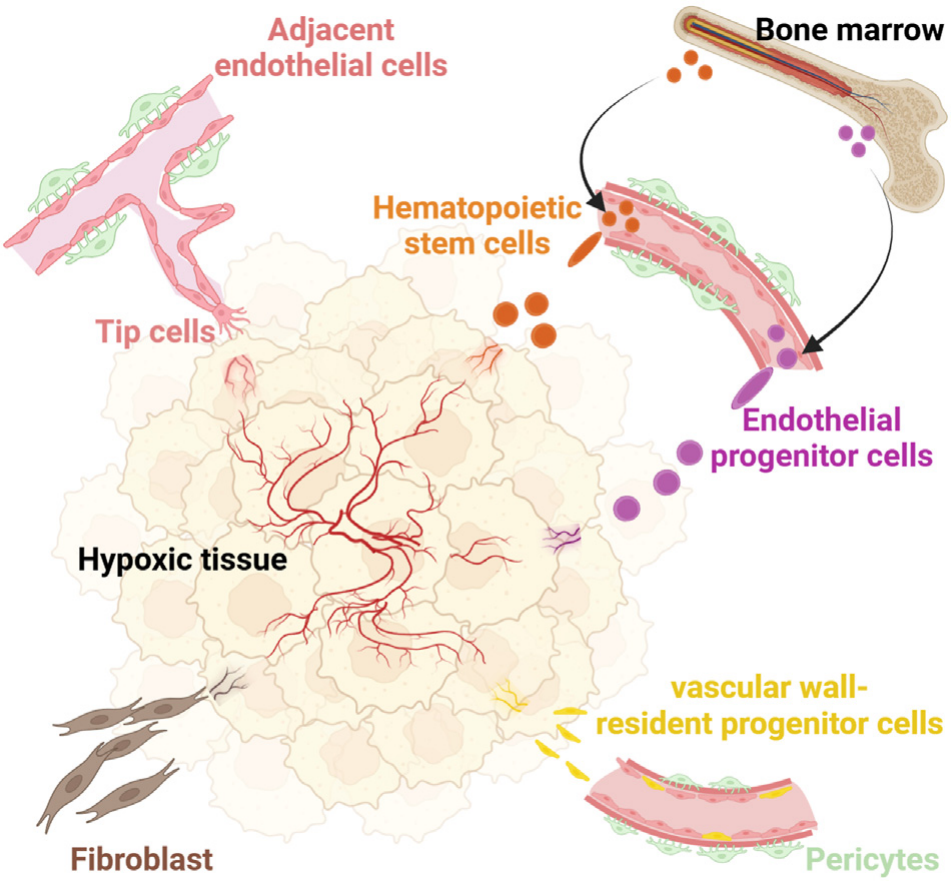
Sources of vascular endothelium in hypoxic angiogenesis. In hypoxic angiogenesis, vascular endothelial cells derive from multiple sources, including the recruitment of adjacent vascular endothelial cells (light pink), differentiation of vascular wall-resident progenitor cells (yellow), differentiation of bone marrow-derived hematopoietic stem cells (orange), recruitment of bone marrow-derived endothelial progenitor cells (purple), and transdifferentiation of fibroblasts (brown).

Multiple cell sources of ECs have been reported in hypoxic angiogenesis in murine models, as illustrated in Figure 1. Adjacent ECs serve as a primary source of vascular ECs. These adjacent ECs secrete angiogenic factors to promote EC proliferation and migration. They also secrete factors crucial for extracellular matrix degradation and basement membrane remodeling, which are essential for lumen-like structure formation.^38^^,^^92^^,^^93^

In addition, vascular wall-resident progenitor cells possess the ability to differentiate into vascular ECs. The CD34^+^CD31^−^ adventitial cells are believed to represent the resident progenitor cells. Studies have shown that these resident progenitor cells can differentiate into ECs both *in vivo* and *in vitro*.^94^^,^^95^

The recruitment of bone marrow-derived cells, such as hematopoietic stem cells (HSCs) and EPCs, also contributes to the vascular EC pool. The c-kit^+^/bone marrow stem cells antigen-1^+^ (Sca-1^+^)/lineage^−^ HSCs are identified as bone marrow-derived HSCs that can differentiate into ECs in bone marrow transplantation models.^96^ In retinal neovascularization models, bone marrow-derived HSCs act as ECs in vascular repair.^97^ Lineage tracing studies and single-cell transcriptomics identify a population of CD133^+^ bone marrow-derived endothelial-like cells as potential EPCs, which contribute to neovascularization *in vivo*.^98^ The fluorescently labeled human EPCs and a specific subtype CD34^+^ EPCs have been shown to integrate into hypoxic tissue sites and participate in neovascularization in irradiated mouse models.^99^ These results are further supported by large-scale single-cell studies.^100^

Additionally, vascular ECs can derive from fibroblast transdifferentiation. Fibroblast lineage tracing murine studies suggest that cardiac fibroblasts can transdifferentiate into ECs, expressing specific markers associated with endothelial identity and forming microvessels *in vivo* in the ischemic cardiac injury mice model. This process is promoted by p53 signaling.^46^^,^^101^ However, in another adult mouse cardiac injury model, lineage tracing murine studies have shown that angiogenesis derives from preexisting ECs.^93^ In a murine ischemic hindlimb model that mimics peripheral arterial disease, lineage tracing studies identify a subset of fibroblasts that can express EC genes and form capillaries. Interestingly, this transdifferentiation process is mediated by innate immune signaling.^102^

Although lymphatic ECs at specific locations have been reported to dedifferentiate into vascular ECs in zebrafish, this phenomenon has not been confirmed in mammalian models.^103^ Besides, a wide spectrum of cells can transform into ECs in *in vitro* conditions. For instance, Sca-1^+^ cells derived from the adventitia can differentiate into ECs, as evidenced by the expression of CD34, CD31, and VE-cadherin 104. Human neural stem cells have been shown to differentiate into vascular ECs via cell fusion mechanisms.^105^ Bone marrow-derived mesenchymal stem cells can be induced to differentiate into vascular ECs.^106^ Similarly, human amniotic membrane cells have been reprogrammed to function as vascular ECs.^107^ Studies in mice suggest that the normal arterial wall contains side population cells with vascular progenitor potential, which can be activated by specific growth or transformation factors.^108^ However, these findings require further validation *in vivo*.

### Origins of ECs in tumor angiogenesis

**Figure 2:**
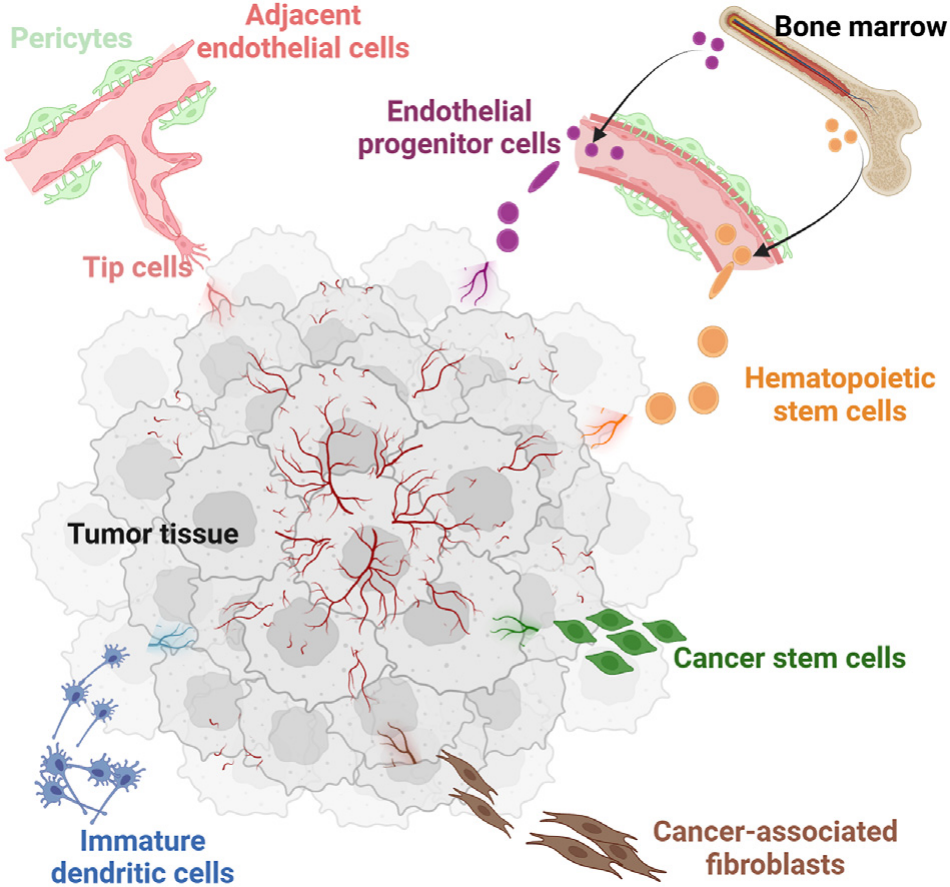
Sources of vascular endothelium in tumor angiogenesis. In tumor angiogenesis, vascular endothelial cells derive from multiple sources, including the recruitment of adjacent vascular endothelial cells (light pink), recruitment of bone marrow-derived endothelial progenitor cells (purple), differentiation of hematopoietic stem cells (orange), differentiation of cancer stem cells (green), transdifferentiation of cancer-associated fibroblasts (brown), and transdifferentiation of immature dendritic cells (blue).

In the context of cancer, angiogenesis takes on a distinct character. Tumor angiogenesis is characterized by unregulated and disorganized blood vessel formation, resulting in abnormal and chaotic vascular networks within the tumor microenvironment. The reported sources of vascular ECs in solid tumors are more diverse than hypoxic angiogenesis, as illustrated in Figure 2.

The primary reported source of tumor vascular ECs involves adjacent ECs. Solid tumors frequently undergo rapid proliferation and division, leading to increased oxygen and nutrient consumption. This heightened metabolic activity results in the release of pro-angiogenic factors such as VEGF, which stimulate adjacent ECs to proliferate and form angiogenic sprouts. These sprouts migrate toward the tumor-driven angiogenic stimulus. As ECs migrate and proliferate, they adhere to form tubular structures that eventually develop into functional lumens. These lumens fuse to establish a new network of blood vessels within the tumor.^109^, ^110^, ^111^, ^112^

Another source involves recruiting bone marrow-derived cells, including EPCs and HSCs. In transgenic mouse models, EPCs have been shown to migrate to tumor sites and contribute to tumor neovascularization.^112^, ^113^, ^114^ In chimeric mouse models, bone marrow cells are recruited to newly formed and remodeled tumor vasculature.^115^ Studies on angiogenic-defective, tumor-resistant Id-mutant mice have shown that transplanting wild-type EPCs restores tumor angiogenesis and growth.^116^ Research using a co-implantation tumor xenograft model has found that EPCs contribute to tumor neovascularization.^117^ Additionally, in mice with brain tumors, bone marrow-derived green fluorescent protein^+^ (GFP^+^) HSCs gave rise to elongated CD34^+^/fetal liver kinase 1^+^ (Flk-1^+^) cells. These HSCs exhibited endothelial morphology and expressed specific endothelial phenotypes.^118^ Overall, these findings suggest that bone marrow-derived cells can differentiate into cells with endothelial characteristics, contributing to blood vessel formation within tumors.

Tumor vascular ECs can originate from the differentiation of cancer stem cells. In kidney cancer, some vessels found in tumors derived from CD105^+^ clones were of human origin, indicating that tumor stem cells possess the ability to differentiate into ECs *in vivo*.^119^ In breast cancer, researchers isolated a population of cancer stem cells capable of forming transplantable tumors, with certain intra-tumoral vessels being of human origin, further confirming that cancer stem cells can differentiate into ECs *in vivo*.^120^ Reports have also shown that glioblastoma stem cells can give rise to ECs.^121^, ^122^, ^123^, ^124^, ^125^, ^126^ However, subsequent studies employing *in vivo* lineage tracing techniques have provided robust evidence that glioblastoma stem cells primarily differentiate into pericytes rather than ECs.^127^

Cancer-associated fibroblasts can transdifferentiate into tumor vascular ECs. A study on pancreatic ductal carcinoma demonstrates that the protein kinase RNA-like endoplasmic reticulum kinase (PERK)-eukaryotic initiation factor 2 alpha (eIF2α)-ERK1/2 signaling pathway facilitates the transdifferentiation of cancer-associated fibroblasts into tumor ECs. These cancer-associated fibroblasts acquire endothelial characteristics and contribute to tumor angiogenesis.^128^ In orthotopic colorectal cancer xenograft mouse models, cancer-associated fibroblasts have been shown to transdifferentiate into tumor vascular ECs, exhibiting endothelial features.^129^

Tumor vascular ECs can also derive from the transdifferentiation of immature dendritic cells. A unique population of dendritic cells has been discovered in mouse and human ovarian carcinomas. These dendritic cells typically express endothelial markers, including CD34 and VE-cadherin, and have the capacity to form blood vessel-like structures both *in vitro* and *in vivo*. These findings suggest that immature dendritic cells promote tumor growth and exhibit endothelial-like functions depending on the microenvironment. Moreover, they prevent the initiation of an immune response against tumor antigens.^130^

In summary, tumor angiogenesis involves the generation of ECs from diverse cell types, resulting in a variety of tumor vascular EC characteristics. Although not fully understood, it can be assumed that these vascular ECs exhibit distinct angiogenic features and responses to major angiogenic signals. These unique properties significantly impact their response to anti-angiogenic therapies, presenting both challenges and opportunities in cancer treatment. An in-depth understanding of the mechanisms driving this process is crucial for advancing the development of more effective anti-angiogenic therapies for cancer.

### Application of anti-angiogenic agents and adverse events in cancer

Tumor angiogenesis is triggered by the activation of vascular ECs, often driven by hypoxia within the tumor microenvironment. In response, tumor cells secrete various angiogenic factors that significantly stimulate the activation of vascular ECs. Since ECs are crucial for the formation and maintenance of tumor vasculature, targeting ECs with anti-angiogenic therapy is an effective strategy to impede tumor growth and prevent metastasis. Over the years, numerous pro- and anti-angiogenic factors have been identified.^30^^,^^52^^,^^131^^,^^132^ This ongoing research has broadened our understanding of the molecular mechanisms underlying angiogenesis and has promoted the development of various anti-angiogenic therapies.

Currently, anti-angiogenic agents used in clinical treatment can be categorized into two primary groups: small-molecule multi-target inhibitors and large-molecule inhibitors. The latter also includes endogenous pan-target inhibitors of angiogenesis. Both categories function by disrupting signaling pathways that promote the formation of new blood vessels in tumors.

Small-molecule multi-target inhibitors are the most common anti-angiogenic agents available in the market. Currently, nineteen orally administered inhibitors are approved for global use. Imatinib targets PDGFR, c-KIT, and Abl, and is used clinically for hematologic neoplasms^133^ and metastatic gastrointestinal stromal tumors.^134^ Gefitinib is a specific inhibitor that targets epidermal growth factor receptor (EGFR), insulin-like growth factor (IGF), and PDGF, clinically applied for non-small cell lung cancer (NSCLC).^135^ Sorafenib and sunitinib effectively inhibit various tyrosine kinases such as FMS-like tyrosine kinase 3 (FLT3), KIT, and RET, suppressing tumor growth and angiogenesis by inhibiting VEGFR2/3 and PDGFRβ. These agents are effective in treating liver cancer,^136^ renal cell carcinoma,^137^ NSCLC,^136^ thyroid cancer,^138^^,^^139^ and gastrointestinal cancers, including pancreatic cancer.^140^ Apatinib binds to VEGFR2, RET, and c-KIT, suppressing blood vessel formation, and is primarily used for advanced or metastatic gastric cancer.^141^ Erlotinib, lapatinib, afatinib, and neratinib all bind to EGFR and human epidermal growth factor receptor 2 (HER2), inhibiting downstream phosphorylation and angiogenesis, and are clinically used for NSCLC,^142^^,^^143^ pancreatic cancer,^144^ and breast cancer.^145^, ^146^, ^147^ Cabozantinib and regorafenib both target VEGFR, TIE2, c-KIT, and RET. Clinically, they are employed for the treatment of renal cell carcinoma and thyroid cancer,^148^^,^^149^ as well as gastrointestinal stromal tumors and hepatocellular carcinoma.^150^, ^151^, ^152^ Axitinib and nintedanib bind to targets such as VEGFR, PDGFR, and c-KIT. These drugs have been clinically used for the treatment of advanced renal cell carcinoma,^153^ colorectal cancer,^154^^,^^155^ ovarian cancer,^156^ and other malignancies.^157^ Targeting PDGFR, VEGFR, and FGFR to effectively suppress angiogenesis renders pazopanib and anlotinib valuable in the treatment of osteosarcoma,^158^ breast cancer,^159^ ovarian cancer,^160^ renal cancer,^161^ soft tissue sarcoma, and lung cancer.^162^ Vandetanib and lenvatinib are used in the treatment of thyroid cancer,^163^^,^^164^ NSCLC,^165^ and hepatocellular carcinoma^166^ due to their ability to inhibit angiogenesis by binding to targets such as FGFR, VEGFR, and RET. Fruquintinib effectively inhibits angiogenesis by binding to the VEGFR, FGFR1, and RET targets and is primarily used for metastatic colorectal cancer.^167^ Erdafitinib exerts angiogenesis suppression through its binding to key targets, including FGFR, PDGFR, and VEGFR2, exhibiting promising outcomes in the treatment of metastatic urothelial carcinoma.^168^ In summary, these multi-target anti-angiogenic agents have shown significant therapeutic efficacy in the treatment of various malignant tumors, demonstrating their importance in cancer therapy (Table 1).

**Table 1:** Small molecule multi-target anti-angiogenic drugs approved by FDA for clinical treatment.

Small molecule multi-target angiogenesis inhibitors				
Drug	Description	Target(s)	FDA Approval Cancer Type (Clinical Trials)	Cancer Type (Clinical Trials and Stage)
Imatinib	mRTKs inhibitor	PDGFR, c-KIT, Abl	Ph^+^ CML-(NCT00219739), Ph^+^ ALL-(NCT00376467), GIST-(NCT01151852), DFSP-(NCT00122473)	
Gefitinib	mRTKs inhibitor	EGFR, IGF, PDGF	NSCLC-(NCT00770588)	
Erlotinib	mRTKs inhibitor	EGFR, HER2	NSCLC-(NCT00036647), PAAD-(NCT00040183)	
Sorafenib	mRTKs inhibitor	VEGFR, PDGFRβ, KIT, FLT3, RAF	HCC-(NCT00105443), RCC-(NCT00073307), DTC-(NCT00984282)	AIPC-(NCT00090545 Phase II), NSCLC-(NCT00101413 Phase II)
Sunitinib	mRTKs inhibitor	PDGFR, VEGFR, KIT, FLT3, CSF-1R, RET	GIST-(NCT00075218), RCC-(NCT00083889), PNETs-(NCT00428597)	DTC-(NCT00668811 Phase II), ACC- (NCT00453895 Phase II)
Lapatinib	mRTKs inhibitor	EGFR, HER2	HER2+eBC-(NCT00374322)	
Apatinib	mRTKs inhibitor	VEGFR2, RET, c-KIT	AGC-(NCT01512745)	HCC-(NCT02329860 Phase III), (non)-TNBC-(NCT01653561) and (NCT01176669 Phase II), rCRC-(NCT01287962 Phase III), NSCLC-(NCT03190616 Phase III), DTC-(NCT02731352 PhaseII)
Pazopanib	mRTKs inhibitor	VEGFR, PDGFR, FGFR1/3, KIT, ITK, LCK, c-FMS	RCC-(NCT00387764), STS-(NCT00753688)	NSCLC-(NCT00367679 Phase II), EOC/FTC/PPC-(NCT00866697 Phase II)
Vandetanib	mRTKs inhibitor	EGFR, VEGFR, RET, BRK, TIE2, EPHR, SFKs	MTC-(NCT00410761)	
Axitinib	mRTKs inhibitor	VEGFR, PDGFRβ	RCC-(NCT00282048) and (NCT00678392)	mRCC-(NCT02579811 Phase II), TC-(NCT00389441 Phase II)
Regorafenib	mRTKs inhibitor	RET, VEGFR, KIT, PDGFR, FGFR1/2, TIE2, DDR2, Eph2A, RAF1, SAPK2, PTK5, Abl, CSF1R	mCRC–(NCT01103323), HCC-(NCT01271712), GIST-(NCT01774344)	RCC-(NCT00664326 Phase II)
Cabozantinib	mRTKs inhibitor	MET, VEGFR, AXL, RET, ROS1, TYRO3, MER, KIT, TRKB, FLT3, TIE2	RCC-(NCT01865747), HCC-(NCT01908426), DTC-(NCT03690388)	NF1-PN-(NCT02101736 Phase II), mUC-(NCT01688999 Phase II)
Afatinib	mRTKs inhibitor	EGFR, HER2	NSCLC-(NCT00949650), sq-NSCLC-(NCT01523587)	HNSC-(NCT01427478 Phase III)

Nintedanib	mRTKs inhibitor	PDGFR, VEGFR, FGFR, CSF1R, FLT3, SFKs	IPF-(NCT01335464), PF-ILD-(NCT02999178), SSC-ILD-(NCT02597933)	
Lenvatinib	mRTKs inhibitor	VEGFR1/2/3, FGFR, PDGFRα, KIT, RET	DTC-(NCT01321554), HCC-(NCT01761266)	BTC-(NCT02579616 Phase II), RET + LUAD-(NCT01877083 Phase II)
Neratinib	mRTKs inhibitor	EGFR, HER1/2/4	HER2+ eBC-(NCT00878709)	
Anlotinib	mRTKs inhibitor	VEGFR, PDGFR, FGFR, c-Kit, RET	NSCLC-(NCT02388919), SCLC-(NCT03059797), STS-(NCT02449343)	mRCC-(NCT02072044 Phase II), MTC-(NCT02586350 Phase II)
Fruquintinib	mRTKs inhibitor	VEGFR, FGFR1, RET	mCRC-(NCT04322539)	NSCLC-(NCT02590965 Phase II)
Erdafitinib	mRTKs inhibitor	FGFR, RET, CSF1R, PDGFR, FLT4, KIT, VEGFR2	mUC-(NCT02365597)	AST-(NCT04083976 Phase II)

Ph^+^ CML, Philadelphia chromosome positive chronic myeloid leukemia; Ph^+^ ALL, Philadelphia chromosome positive acute lymphoblastic leukemia; GIST, gastrointestinal stromal tumors; DFSP, unresectable, recurrent and/or metastatic dermatofibrosarcoma protuberans; NSCLC, non-small cell lung cancer; PAAD, locally advanced, unresectable or metastatic pancreatic cancer; HCC, hepatocellular carcinoma; RCC, advanced renal cell carcinoma; DTC, differentiated thyroid carcinoma; AIPC, metastatic, androgen-independent prostate cancer; PNETs, advanced pancreatic neuroendocrine tumors; ACC, refractory adrenocortical carcinoma; HER2^+^ eBC, early-stage HER2-positive breast cancer; AGC, advanced or metastatic gastric cancer; non-TNBC, non-triple-negative metastatic breast cancer; TNBC, metastatic triple-negative breast cancer; rCRC, refractory colorectal cancer; STS, advanced soft tissue sarcoma (except for gastrointestinal stromal tumors and adipocytic sarcoma); EOC/FTC/PPC, ovarian, fallopian tube, or primary peritoneal cancer; MTC, unresectable, locally advanced or metastatic medullary thyroid cancer; mRCC, metastatic renal cell carcinoma; mCRC, metastatic colorectal cancer; NF1-PN, neurofibromatosis type 1-associated plexiform neurofibromas; mUC, platinum-refractory metastatic urothelial carcinoma; sq-NSCLC, metastatic squamous non-small cell lung cancer; HNSC, squamous cell carcinoma of the head and neck; IPF, idiopathic pulmonary fibrosis; PF-ILD, progressive fibrosing interstitial lung disease; SSC-ILD, systemic sclerosis-associated interstitial lung disease; BTC, biliary tract cancer; RET^+^ LUAD, RET fusion-positive lung adenocarcinoma; SCLC, small cell lung cancer; AST, advanced solid tumors with FGFR alterations.

Large-molecule angiogenesis inhibitors (Table 2) are divided into two subclasses. The first subclass consists of nine monotherapeutic large-molecule angiogenesis inhibitors, including one fusion protein (ziv-aflibercept) and eight monoclonal antibodies (trastuzumab, bevacizumab, cetuximab, panitumumab, pertuzumab, ramucirumab, necitumumab, and olaratumab). Ziv-aflibercept exerts its anti-angiogenic effects by selectively binding to VEGFA/B, making it a common therapeutic option for colorectal cancer treatment.^169^ Trastuzumab and pertuzumab are primarily used in breast cancer treatment due to their high affinity for HER2 binding.^170^^,^^171^ Bevacizumab, the first large molecule targeted anti-tumor drug, specifically targets all subtypes of VEGFA, inhibiting its interaction with endothelial VEGFR and effectively suppressing tumor angiogenesis. It is extensively employed in managing various malignancies, including colorectal cancer,^172^ breast cancer,^173^ and renal cancer.^174^ Cetuximab and panitumumab exhibit potent EGFR binding capabilities, thereby inhibiting tumor angiogenesis. They are commonly used in the clinical treatment of colorectal cancer^175^^,^^176^ and NSCLC.^177^ Ramucirumab specifically binds to VEGFR2, inhibiting angiogenesis, and is primarily employed to treat gastric cancer,^178^ colorectal cancer,^179^ lung cancer,^180^ and hepatocellular carcinoma.^181^ Necitumumab selectively targets EGFR to suppress neovascularization and is clinically utilized for NSCLC therapy.^182^ Olaratumab specifically binds to VEGFR, inhibiting new blood vessel formation, and is primarily used for treating soft tissue sarcoma^183^ and gastrointestinal stromal tumors.^184^

**Table 2:** Large molecule anti-angiogenic drugs approved by FDA for clinical treatment.

Large molecule angiogenesis inhibitors				
Macromolecular single-target angiogenesis inhibitors				
Drug	Description	Target(s)	FDA Approval Cancer Type (Clinical Trials)	Cancer Type (Clinical Trials and Stage)
Trastuzumab	Monoclonal antibody	HER2	HER2^+^ eBC-(NCT00045032), HER2^+^ AGC-(NCT01774786)	OAC-(NCT01196390 Phase III), mCRC-(NCT03384940 Phase II)
Cetuximab	Monoclonal antibody	EGFR	HNSC-(NCT00004227), mCRC-(NCT00079066)	NSCLC-(NCT00148798 Phase III), FL-(NCT00115700 Phase III)
Bevacizumab	Monoclonal antibody	VEGFA	mCRC-(NCT00109070), nsq-NSCLC-(NCT00021060), GBM-(NCT01290939), RCC-(NCT00738530), R/M CC-(NCT00803062), mBC-(NCT00028990), EOC/FTC/PPC-(NCT00976911), mHCC-(NCT03434379)	
Panitumumab	Monoclonal antibody	EGFR	mCRC-(NCT00113763)	RM-HNC-(NCT00460265 Phase III), mCRC-(NCT01412957 Phase III), HER2- IBC-(NCT01036087 Phase II), CHOL-(NCT00948935 Phase II)
Pertuzumab	Monoclonal antibody	HER2	HER2+ mBC-(NCT00567190), HER2+ eBC-(NCT01358877)	
Ziv-aflibercept	Monoclonal antibody	VEGFA/B	mCRC-(NCT00519285)	OV-(NCT00327444 Phase II), PT-SCLC-(NCT00828139 Phase II)
Ramucirumab	Monoclonal antibody	VEGFR2	GC/GEJC-(NCT00917384), HCC-(NCT02435433), NSCLC-(NCT01168973), mCRC-(NCT01183780)	mUC-(NCT02426125 Phase III)
Necitumumab	Monoclonal antibody	EGFR	NSCLC-(NCT00982111)	
Olaratumab	Monoclonal antibody	PDGFRα	STS-(NCT02451943)	GIST-(NCT01316263 Phase II)

Endogenous pan-target angiogenesis inhibitor				

Drug	Description	Target(s)	FDA approval cancer type (clinical trials)	Cancer type (clinical trials and stage)

Endostar	Recombinant human endostatin	VEGFR, VEGFR2, PDGFR, TGF-β, PEDF	NSCLC-(NCT00708812)	MM-(NCT00813449 Phase II), BC-(NCT01479036 Phase III)

HER2^+^ eBC, HER2-positive primary breast cancer; HER2^+^ AGC, HER2-positive advanced gastric cancer; OAC, esophageal adenocarcinoma; mCRC, HER2-expressing metastatic colorectal cancer; HNSC, squamous cell carcinoma of the head and neck; NSCLC, non-small cell lung cancer; FL, early stage follicular lymphoma; nsq-NSCLC, first-line non-squamous non-small cell lung cancer; GBM, recurrent glioblastoma; RCC, metastatic renal cell carcinoma; R/M CC, persistent, recurrent, or metastatic cervical cancer; mBC, locally recurrent or metastatic breast cancer; EOC/FTC/PPC, epithelial ovarian, fallopian tube, or primary peritoneal cancer; mHCC, locally advanced unresectable and/or metastatic hepatocellular carcinoma; RM-HNC, recurrent and/or metastatic head and neck cancer; HER2- IBC, primary HER2-negative inflammatory breast cancer; CHOL, advanced cholangiocarcinoma; HER2+ mBC, HER2-positive metastatic breast cancer; OV, advanced ovarian cancer; PT-SCLC, platinum-treated small-cell lung cancer; GC/GEJC, advanced or metastatic gastric or gastro-esophageal junction adenocarcinoma; HCC, hepatocellular carcinoma; mUC, locally advanced or metastatic urothelial carcinoma; STS, soft tissue sarcoma; GIST, metastatic gastrointestinal stromal tumors; MM, metastatic melanoma; BC, breast cancer.

The second subclass includes one endogenous pan-target angiogenesis inhibitor, Endostar, a modified protein derived from endostatin, an angiogenesis inhibitor. Endostar targets multiple receptors involved in angiogenesis, including VEGFR, VEGFR2, PDGFR, and PEDFR. It has shown promise in clinical applications for various types of cancer, including nasopharyngeal carcinoma,^185^ NSCLC,^186^ cervical cancer,^187^ and ovarian cancer.^188^

### Limitations in anti-angiogenic therapy

The use of angiogenic inhibitors in cancer therapy, which aims to impede tumor growth by halting new blood vessel formation, represents a promising strategy for treating various solid tumors. However, the efficacy of anti-angiogenic therapy is limited by the complex and multifaceted mechanisms underlying tumor angiogenesis, including restricted therapeutic outcomes, drug resistance, and significant adverse events.

Initially, anti-angiogenic therapy was presumed to have lower toxicity compared with traditional chemotherapeutic agents, largely due to the genetic stability and quiescence of ECs under normal physiological conditions and the selectivity of targeted drugs. However, this assumption has proven overly simplistic. In clinical practice, adverse events and patient complications have frequently emerged, significantly compromising therapeutic efficacy.^30^^,^^189^ Current therapies primarily target key angiogenic signaling pathways. Yet, the heterogeneity of EC phenotypes within and across tumors leads to substantial variability in treatment effectiveness, both between different tumor types and among patients.^190^ Consequently, these therapies often affect only the endothelial subpopulations that rely on the targeted pathways, which partially explains the limited efficacy of anti-angiogenic treatments.^190^

Drug resistance remains a critical obstacle that consistently undermines the clinical success of anti-angiogenic therapies. This resistance manifests in two primary forms: intrinsic (congenital) resistance and acquired resistance.^52^ Intrinsic resistance arises from patient- and tumor-specific genetic factors, resulting in inherent insensitivity to anti-angiogenic agents. Acquired resistance, on the other hand, occurs in patients who initially respond to therapy but later develop resistance, leading to disease progression despite treatment. Mechanisms driving acquired resistance include the recruitment of perivascular cells, such as pericytes, which protect immature tumor blood vessels from destruction by anti-angiogenic agents^191^; the up-regulation of compensatory pro-angiogenic signaling pathways, such as hepatocyte growth factor (HGF), bFGF, PlGF, and Dll4^192^, ^193^, ^194^; and the mobilization of bone marrow-derived cells, including EPCs,^195^ pericyte progenitor cells,^196^ and tumor-associated macrophages.^197^ Additionally, drug resistance is influenced by factors such as the tumor microenvironment, endothelial heterogeneity,^45^^,^^198^ autophagy in tumor cells,^199^ stromal cell infiltration,^200^ tumor types,^201^ and other variables, all of which collectively impact patient response and tolerance to anti-angiogenic therapy.

The occurrence of common and severe adverse events during clinical treatment underscores the toxicity and challenges associated with anti-angiogenic therapy. Patients undergoing this treatment may encounter a broad spectrum of adverse events, which can manifest locally at the tumor site or systemically in other organs. Prominent side effects include hypertension,^202^ proteinuria,^203^ gastrointestinal disturbances,^204^ and bleeding complications.^205^ Furthermore, tumors may develop resistance mechanisms such as activating alternative pro-angiogenic pathways or acquiring genetic mutations, which collectively render tumor cells increasingly resistant and unresponsive to treatment.^206^

## Discussion

Although ECs are present in all organs as a single-cell layer, they have traditionally been regarded as a relatively inert cell population. However, recent research has redefined the vascular endothelium as a highly dynamic, interactive, and systemically disseminated organ that plays a critical role in cancer.^207^ A hallmark feature of cancer is its capacity to drive the formation of new blood vessels, a process referred to as tumor angiogenesis.^208^^,^^209^ Anti-angiogenic agents impede tumor growth by disrupting new blood vessel formation,^210^, ^211^, ^212^ often serving as an adjunct to chemotherapy, radiotherapy, and immunotherapy.

The profound mechanistic study of angiogenesis has been instrumental in advancing the development of anti-angiogenic therapy. However, the effectiveness of these treatments varies significantly across tumors and patients, largely due to the inter- and intra-tumoral heterogeneity in EC phenotypes.^190^ Current therapy primarily aims to inhibit key angiogenic signaling pathways, yet this specificity often limits their impact to only the endothelial subpopulations that rely heavily on these pathways. This selectivity may contribute to the limited efficacy and resistance commonly observed in anti-angiogenic therapy.^190^

A more comprehensive understanding of the tumor vascular endothelial population, including their origins, responsive angiogenic mechanisms, and functions, will provide critical insights for improving therapeutic strategies. Furthermore, identifying molecular distinctions between tumor vasculature and normal blood vessels could pave the way for the development of more precise and effective therapies that specifically target tumor-associated vasculature.

## CRediT authorship contribution statement

**Yulong Han:** Writing – original draft, Visualization, Validation, Software, Project administration, Formal analysis, Data curation, Conceptualization. **Binqiang Zhu:** Resources, Methodology, Investigation, Formal analysis, Data curation. **Shu Meng:** Writing – review & editing, Writing – original draft, Visualization, Validation, Formal analysis, Data curation, Conceptualization.

## Funding

This study is supported by research grants from the 10.13039/501100001809National Natural Science Foundation of China (No. 82170512) and the Major Project of Guangzhou National Laboratory of China (No. GZNL2023A02009) to Shu Meng.

## Conflict of interests

The authors declared no conflict of interests.
